# Notch Signaling Regulates the Function and Phenotype of Dendritic Cells in *Helicobacter pylori* Infection

**DOI:** 10.3390/microorganisms11112818

**Published:** 2023-11-20

**Authors:** Qiaoyuan Liu, Chuxi Chen, Yunxuan He, Wenhao Mai, Shipeng Ruan, Yunshan Ning, Yan Li

**Affiliations:** School of Laboratory Medicine and Biotechnology, Southern Medical University, No. 1023, South Shatai Road, Baiyun District, Guangzhou 510515, Chinamwh1617552870@163.com (W.M.);

**Keywords:** *Helicobacter pylori*, dendritic cells, Notch signaling, CD4^+^ T cells

## Abstract

Notch signaling manipulates the function and phenotype of dendritic cells (DCs), as well as the interaction between DCs and CD4^+^ T cells. However, the role of Notch signaling in *Helicobacter pylori* (*H. pylori*) infection remains elusive. Murine bone marrow-derived dendritic cells (BMDCs) were pretreated in the absence or presence of Notch signaling inhibitor DAPT prior to *H. pylori* stimulation and the levels of Notch components, cytokines and surface markers as well as the differentiation of CD4^+^ T cells in co-culture were measured using quantitative real-time PCR (qRT-PCR), Western blot, enzyme-linked immunosorbent assay (ELISA) and flow cytometry. Compared with the control, the mRNA expression of all Notch receptors and Notch ligands Dll4 and Jagged1 was up-regulated in *H. pylori*-stimulated BMDCs. The blockade of Notch signaling by DAPT influenced the production of IL-1β and IL-10 in *H. pylori*-pulsed BMDCs, and reduced the expression of Notch1, Notch3, Notch4, Dll1, Dll3 and Jagged2. In addition, DAPT pretreatment decreased the expression of maturation markers CD80, CD83, CD86, and major histocompatibility complex class II (MHC-II) of BMDCs, and further skewed Th17/Treg balance toward Treg. Notch signaling regulates the function and phenotype of DCs, thus mediating the differentiation of CD4^+^ T cells during *H. pylori* infection.

## 1. Introduction

*Helicobacter pylori* (*H. pylori*) is a micro-aerobic, spiral-shaped Gram-negative bacterium that mainly colonizes human gastric mucosa and easily causes various gastrointestinal diseases such as gastritis, peptic ulcers and even gastric cancer. *H. pylori* was listed as Class I carcinogen by World Health Organization in 1994 [1]. At present, over 50% of the world’s population is infected with *H. pylori* [2]. Although the development of antibiotics combined with proton pump inhibitors has achieved an excellent therapeutic effect in controlling infection, drug resistance and immune escape [3] remain two major problems in the clearance of *H. pylori*. In general, *H. pylori* infection induces a robust immune response in the host, but this process is not enough to completely eliminate the pathogen, and can even lead to lifelong infection. Thus, the prevention and treatment of *H. pylori* infection is still a major public health problem to be settled urgently. Therefore, it is necessary to explore the pathogenesis and immune response mechanisms in *H. pylori* infection and develop novel therapeutic strategies for controlling the infection.

Innate immunity is the first line of defense against *H. pylori*. It has been acknowledged that dendritic cells (DCs) are key members of innate immunity and the most powerful antigen-presenting cells (APCs) that act as messengers between innate and adaptive immune responses [4]. Several studies have demonstrated that DCs play an essential role in the immune responses caused by *H. pylori* infection [5,6]. The interaction between DCs and CD4^+^ T cells induced CD4^+^ T cells differentiation into Th1, Th2, Th17 and Regulatory T cells (Tregs), which determine the progress and prognosis of diseases [7,8]. Furthermore, cytokines, such as IL-6, IL-8, IL-12 and IL-23 produced by DCs, were up-regulated after *H. pylori* stimulation, which formed a micro-environment that influenced the differentiation of CD4^+^ T cells [9,10,11]. In addition, compared with uninfected individuals, a large amount of immune-tolerogenic DCs were detected in samples from *H. pylori*-infected mice and human gastric mucosa [3,12]. Collectively, *H. pylori* affects the immune responses of CD4^+^ T cells via altering the function and phenotype of DCs in order to survive. However, the mechanism underlying this process has not been fully elucidated. Therefore, it is of great significance to explore the changes in DC function and phenotype during *H. pylori* infection, laying the foundation for further studies on the mechanism through which DCs regulates CD4^+^ T cell differentiation into different T helper (Th) cell subtypes.

Accumulating evidence has proved that Notch signaling is involved in the activation and regulation of immune cells in various diseases [13,14]. Notch signaling is a key player in modulating the function and phenotype of DCs as well as the interaction between DCs and CD4^+^ T cells [15,16]. In mammals, Notch family consists of four Notch receptors (Notch1–Notch4) and five ligands (Delta Like 1 (Dll1), Dll3, Dll4, Jagged1 and Jagged2) [17]. The binding of ligands to receptors triggers proteolytic cleavage of Notch receptors by γ-secretase, and Notch intracellular domains (NICDs) are subsequently released into cytoplasm, and then migrate into the nucleus to form a transcriptional complex that activates downstream target genes, thus regulating cell proliferation, differentiation, fate determination, diseases development and so on [17,18]. Previous studies showed that Notch ligands Dll1, Dll4 and Jagged1 were highly expressed in DCs [19,20,21], and Notch signaling was essential in DC maturation and DC-mediated T cell response [22,23]. Moreover, Notch ligands Dll1 and Jagged1 altered the phenotype of DCs and affected the secretion of cytokines and chemokines by DCs [24]. Another study revealed that the Delta-like ligands promoted Th1 or Th17 cell polarization, while the Jagged ligands induced Treg or Th2 cell differentiation [18]. Our previous study confirmed that Notch1 was involved in regulating CD4^+^ T cell differentiation into Th1 cells, a subtype considered to be protective in *H. pylori*-infected patients [25]. In addition, Jagged1 augmented the anti-*H. pylori* activity of macrophages by increasing the production of pro-inflammatory factors [26]. However, the detailed mechanisms through which Notch signaling regulates the function and phenotype of DCs and further affects the interaction with CD4^+^ T cells have not been reported.

In this study, we demonstrated that *H. pylori* induced the expression of all Notch receptors and Notch ligands Dll4 and Jagged1 in BMDCs. Blockade of Notch signaling with γ-secretase inhibitor (N-[N-(3,5-Difluorophenacetyl)-L-alanyl]-S-phenylglycine t-butyl ester, DAPT) prior to *H. pylori* infection influenced the expression of Notch components and the production of cytokines in BMDCs. Besides, DAPT pretreatment decreased the expression of maturation markers CD80, CD83, CD86, and MHC-II of BMDCs, and further skewed the Th17/Treg balance toward Treg. Overall, our findings suggested that Notch signaling regulated the function and maturation of DCs and influenced the differentiation of CD4^+^ T cells during *H. pylori* infection. These data will provide insight for elucidating immune defense mechanisms in *H. pylori* infection and developing novel therapies for controlling the infection.

## 2. Materials and Methods

### 2.1. H. pylori Culture

*H. pylori* Sydney strain (SS1) was cultured on *H. pylori* agar basal medium containing 7% sheep blood, supplemented with 0.33 μg/mL polymyxin B sulfate, 5 μg/mL amphotericin B, 5 µg/mL trimethoprim, 5 μg/mL cefsulodin sodium and 10 μg/mL vancomycin hydrochloride at 37 °C in a micro-aerobic environment for 48 h, and then bacterial colonies were collected and resuspended with sterilized phosphate-buffered saline (PBS) for subsequent experiments.

### 2.2. Generation of Murine Bone Marrow-Derived DCs

Female C57BL/6 mice (6–8 weeks old) were provided by the Laboratory Animal Center, Southern Medical University. Bone marrow cells isolated from the femora and tibiae of mice were cultured in RPMI-1640 medium supplemented with 20 ng/mL murine GM-CSF (Peprotech, Rocky Hill, NJ, USA), 10% fetal bovine serum (Gibco, Grand Island, NY, USA), 100 U/mL penicillin, and 100 μg/mL streptomycin to obtain bone marrow-derived DCs (BMDCs) as previously described [27].

### 2.3. Stimulation of BMDCs with H. pylori

BMDCs were seeded on 6-well plates at a density of 1 × 10^6^/mL and cultured in fresh medium supplemented with 10% fetal bovine serum at 37 °C in 5% CO_2_. *H. pylori* was directly added into the medium at a multiplicity of infection (MOI) of 50 CFU/cell as previously described [28], and incubated with BMDCs for 24 h. In the control group, an equivalent volume of PBS was added into the uninfected wells. To block Notch signaling, BMDCs were pretreated with DAPT (20 μM) or equal volume of DMSO for 24 h before *H. pylori* stimulation.

### 2.4. Co-Culture of BMDCs and CD4^+^ T Cells

According to the methods of co-culture described previously [28,29,30], splenic CD4^+^ T cells from syngeneic C57BL/6 mice were selected using anti-CD4-MicroBeads (Miltenyi, Bergisch Gladbach, Germany). BMDCs treated as above were harvested and in the presence of Mitomycin C (20 μg/mL) for 1.5 h followed by washing twice to remove bacteria. Then, BMDCs were collected and co-cultured with CD4^+^ T cells at a ratio of 1:10 for 72 h.

### 2.5. RNA Extraction and Quantitative Real-Time Polymerase Chain Reaction (qRT-PCR)

Total RNA was extracted from cells using TRIzol (Takara, Dalian, China). Complementary DNA (cDNA) was generated from 1 μg of total RNA using a cDNA synthesis kit (EZBioscience, Roseville, MN, USA). qRT-PCR was performed to detect target genes. Amplification was performed in a 10 μL reaction volume under the conditions of one cycle of initial denaturation at 95 °C for 5 min, 95 °C for 10 s and 60 °C for 30 s, followed by 40 cycles to amplify target genes using 2× SYBR Green Color qTR-PCR Mix (EZbiosicence, Roseville, MN, USA).Cycle thresholds obtained were normalized to β-Actin, and the detection of target genes was conducted in triplicate. The relative expression of target genes was calculated using the comparative threshold cycle (Ct) method, 2^−ΔΔCT^ [31]. The sequences of primers are listed in Table 1.

### 2.6. Protein Extraction and Western Blot

Western blot was performed to measure the protein level of Notch ligands Dll1, Dll4 and Jagged1 according to a previous study [32]. Total protein was extracted from cells using RIPA lysis (Genstar, Beijing, China) containing 1 mM Phenylmethylsulfonyl fluoride (PMSF). Subsequently, the concentration of total protein was measured using a Bicinchoninic Acid Kit (Fdbio science, Hangzhou, China). Protein samples were loaded onto sodium dodecyl sulfate-polyacrylamide gel electrophoresis (SDS-PAGE) at 20 μg, and then transferred onto polyvinylidene difluoride (PVDF) membranes (MerckMillipore, Burlington, MA, USA). The primary and secondary antibodies are listed in Table 2. Finally, Super ECL Detection Reagent (Yeasen Biotechnology Co., Ltd., Shanghai, China) was added to the PVDF membranes, and the protein bands were detected with a visualizer.

### 2.7. ELISA

Referring to the method described previously [33], culture supernatants of BMDCs were collected and assayed for IL-1β, IL-6, IL-12p70, TNF-α, IL-10, and TGF-β using ELISA kits (CUSABIO, Wuhan, China) according to the manufacturer’s instructions.

### 2.8. Flow Cytometry

Flow cytometry was used to assess the expression of DC surface markers including CD11c, MHC-II, CD80, CD83, CD86 and Jagged1. BMDCs were resuspended with PBS, treated with FcR blocking reagent, and incubated at 4 °C for 15 min in the dark. Subsequently, BMDCs were incubated with antibodies (BD Biosciences & Biolegend, San Diego, CA, USA) against the surface markers, as above, at 4 °C for 30 min. Finally, samples were detected with a flow cytometer and analyzed using FlowJo vX software.

### 2.9. Statistical Analysis

The data were analyzed using GraphPad Prism 6.0 software and presented as mean ± SD (mean ± SD, *n* = 3). All experiments were repeated at least three times for each treatment group. Comparisons between groups were assessed using a two-sample Student’s *t*-test. Differences were considered statistically significant when *p* values < 0.05 (* *p* < 0.05, ** *p* < 0.01, *** *p* < 0.001, **** *p* < 0.0001, N.S: no statistical difference).

## 3. Results

### 3.1. H. pylori Induced the Expression of All Notch Receptors and Notch Ligands Dll4 and Jagged1 in BMDCs

**Figure 1:**
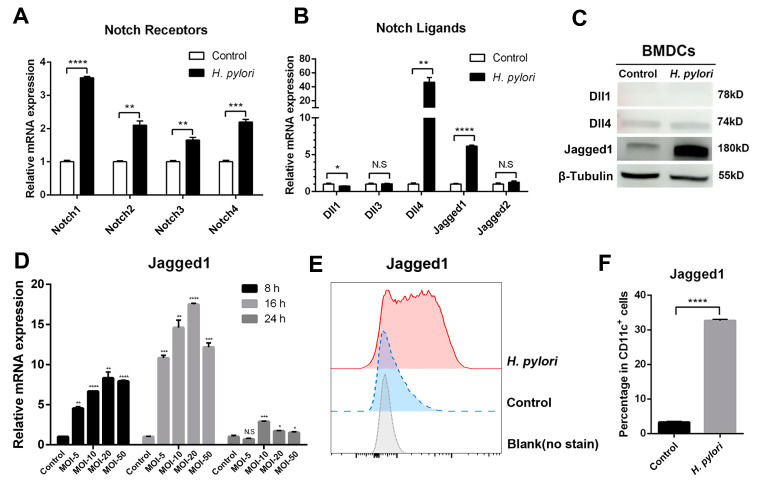
Expression profile of Notch molecules in BMDCs during *H. pylori* infection. BMDCs were stimulated with *H. pylori* (MOI 50) for 24 h. PBS was used as control for *H. pylori*. qRT-PCR was performed to assess the mRNA expression of (**A**) Notch receptors and (**B**) Notch ligands in BMDCs. Relative expression is normalized to β-Actin. (**C**) Western blot was performed to assess the protein levels of Dll1, Dll4 and Jagged1 in BMDCs. β-Tubulin was used as loading control. (**D**) BMDCs were stimulated with different MOI (5, 10, 20 and 50) of *H. pylori* for 8 h, 16 h or 24 h, and qRT-PCR was performed to assess the mRNA level of Jagged1. Flow cytometry was performed to measure the level of Jagged1 on BMDCs stimulated with *H. pylori* (MOI 20) for 16 h, and (**E**) the representative histogram plot and (**F**) the percentage were illustrated. The data are presented as the mean ± SD of three independent experiments. * *p* < 0.05, ** *p* < 0.01, *** *p* < 0.001, **** *p* < 0.0001, N.S: no statistical difference.

Our previous studies demonstrated that Notch1 was involved in Th1 cell differentiation [25] and Jagged1 enhanced macrophage bactericidal activity in response to *H. pylori* infection [26]. To further extend the results, we measured the expression of Notch components in *H. pylori*-stimulated BMDCs using qRT-PCR. It was observed that all Notch receptors were highly expressed (Figure 1A). As for Notch ligands, *H. pylori* significantly induced the expression of Dll4 and Jagged1 at the mRNA level, and slightly decreased Dll1, but there were no statistical differences in the genes of Dll3 and Jagged2 (Figure 1B). For DCs, scientists focus on Notch ligands, and recent studies have proved that Dll1, Dll4, and Jagged1 are involved in activating DCs [34]. Therefore, we detected the protein expression of Dll1, Dll4 and Jagged1 using Western blot. Only Jagged1 was increased remarkably, while Dll1 and Dll4 were not (Figure 1C). Subsequently, we further examined the mRNA expression of Jagged1 in *H. pylori*-infected BMDCs at different time points and MOI. The expression of Jagged1 was the highest at 16 h and MOI 20. Likewise, the level and percentage of Jagged 1 were elevated, as determined using flow cytometry (Figure 1E,F), while Dll4 was too low to be detected. These results indicate that the expression of Notch components in DCs has been altered by *H. pylori*, and Jagged1 may play an essential role.

### 3.2. Notch Signaling Was Involved in the Regulation of Cytokines in H. pylori-Infected BMDCs

**Figure 2:**
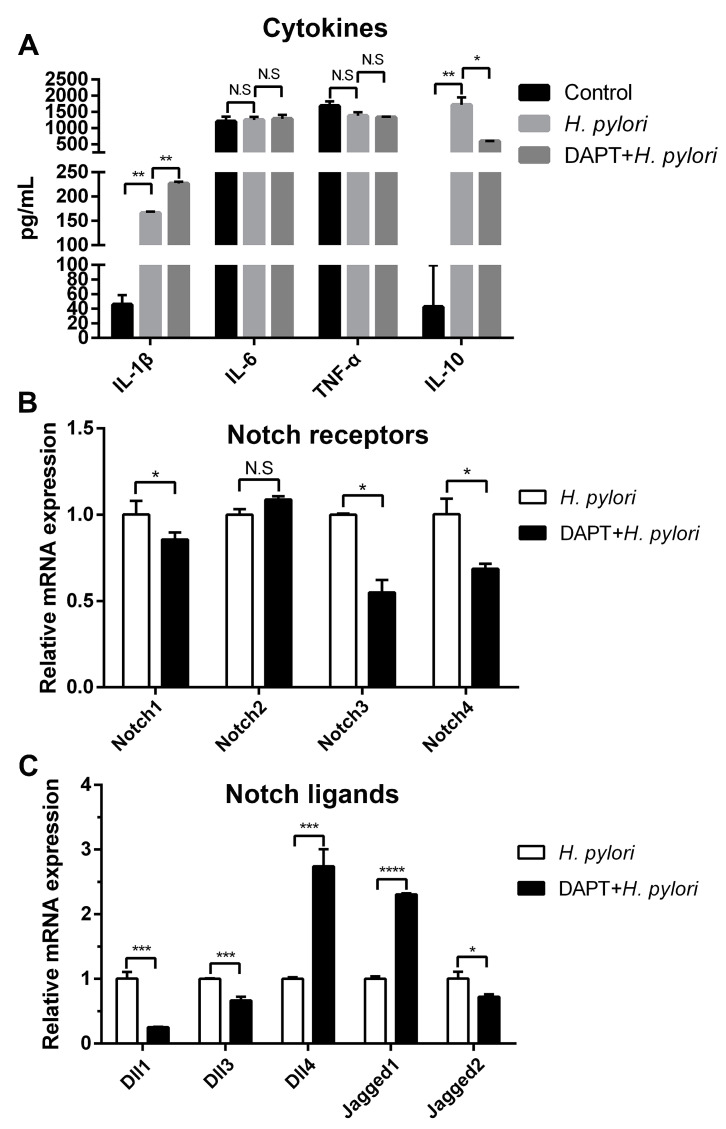
Inhibition of Notch signaling influenced the production of cytokines in BMDCs during *H. pylori* infection. BMDCs were pretreated with DAPT (20 μM) for 24 h, and then stimulated with *H. pylori* (MOI 50) for 24 h. DMSO was used as control for DAPT. (**A**) ELISA was used to determine the level of IL-1β, IL-6, TNF-α and IL-10. qRT-PCR was performed to examine the mRNA expression of (**B**) Notch receptors and (**C**) Notch ligands. Relative expression is normalized to β-Actin. The data are presented as the mean ± SD of three independent experiments. * *p* < 0.05, ** *p* < 0.01 and *** *p* < 0.001, **** *p* < 0.0001, N.S: no statistical difference.

As one of the first defenders, DCs influence the local immune micro-environment via secreting cytokines, thus regulating innate and adaptive immune responses. Meanwhile, the expression of cytokines could be affected by Notch components [35,36]. To explore whether Notch signaling is involved in the expression of cytokines in *H. pylori*-pulsed BMDCs, cells were pretreated in the absence or presence of Notch signaling inhibitor DAPT, and the expression of pro-inflammatory and anti-inflammatory cytokines was determined using ELISA. It was observed that *H. pylori* induced the up-regulation of IL-1β and IL-10, and DAPT treatment increased IL-1β but decreased IL-10 (Figure 2A). Since the above experiments have demonstrated that *H. pylori* induced a change in Notch receptors and ligands in BMDCs, we further detected whether DAPT influences the profile of Notch components using qRT-PCR. It was observed that DAPT pretreatment in BMDCs prior to *H. pylori* stimulation decreased the levels of Notch1, Notch3, and Notch4, as well as Dll1, Dll3 and Jagged2, whereas Dll4 and Jagged1 were still on the rise (Figure 2B,C). This may explain why DAPT treatment leads to changes in the diversity of cytokine levels. Taken together, the results suggest that Notch signaling is involved in the regulation of cytokines, and different Notch components may exert specific effects in BMDCs during *H. pylori* infection.

### 3.3. Notch Signaling Was Involved in Regulating the Phenotype of BMDCs during H. pylori Infection

**Figure 3:**
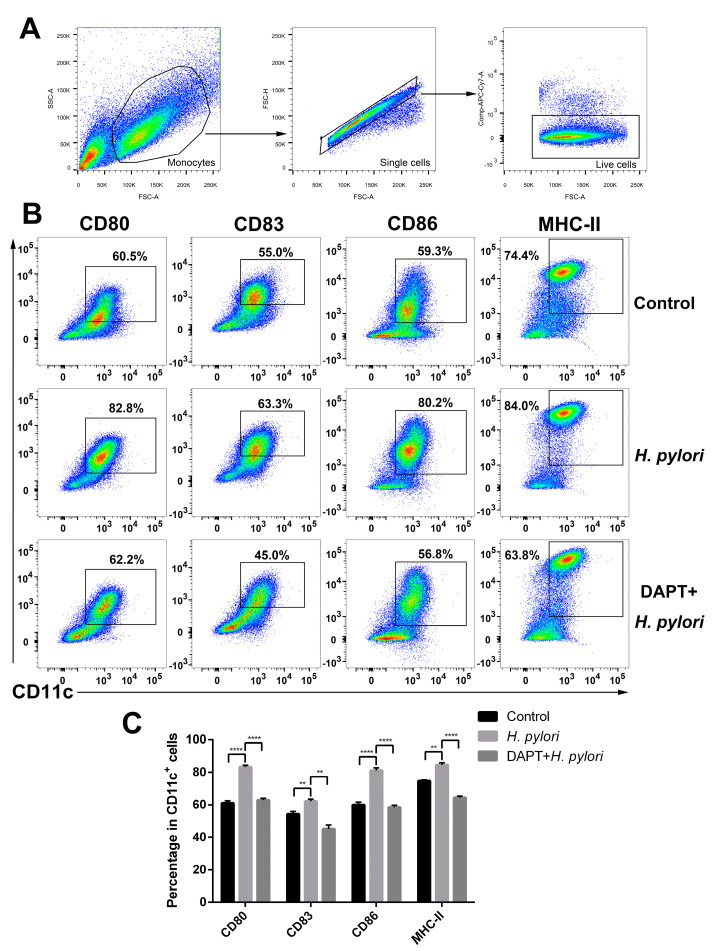
Inhibition of Notch signaling decreased the expression of maturation markers on BMDCs during *H. pylori* infection. BMDCs were pretreated with DAPT (20 μM) for 24 h, and then stimulated with *H. pylori* (MOI 50) for another 24 h. Naïve BMDCs were used as uninfected controls. DMSO was used as a control for DAPT. (**A**,**B**) Representative dot plots and the gating strategy of CD11c^+^ BMDCs are shown. Flow cytometry was performed to evaluate the expression of CD80, CD83, CD86 and MHC-II on BMDCs. (**C**) Percentages of BMDCs expressing CD80, CD83, CD86 or MHC-II are shown. The data are presented as the mean ± SD of three experiments. ** *p* < 0.01 and **** *p* < 0.0001.

The expression of maturation markers is up-regulated in tissue-resident immature DCs upon pathogen stimulation [37]. To verify the effect of Notch signaling on the phenotype of DCs in *H. pylori* infection, BMDCs were pretreated with or without DAPT prior to *H. pylori* stimulation, and surface markers were determined using flow cytometry. *H. pylori* induced the elevation of CD80, CD83, CD86, and MHC-II, whereas DAPT pretreatment resulted in a sharp decrease in those four surface markers (Figure 3), indicating that Notch signaling influences the maturation and phenotype of DCs during *H. pylori* infection.

### 3.4. Inhibition of Notch Signaling in BMDCs Shifted the Th17/Treg Balance toward Treg of CD4^+^ T Cells

**Figure 4:**
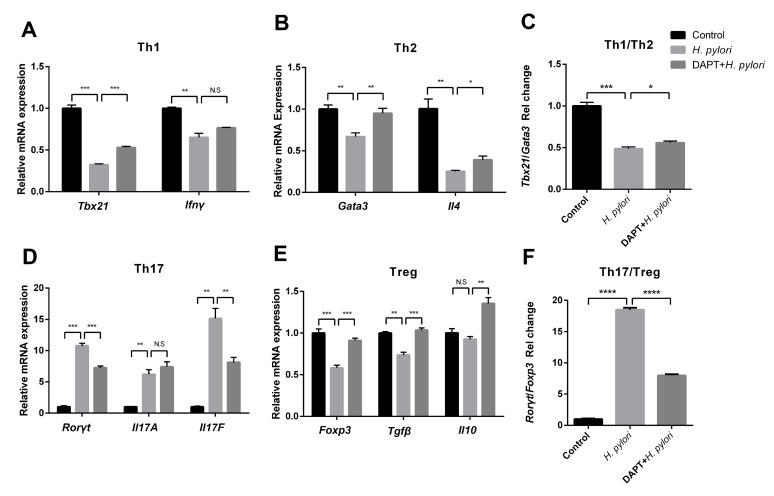
The mRNA expression profile of Th cells was reversed by DAPT in the co-culture system of *H. pylori*-infected BMDCs and CD4^+^ T cells. BMDCs pretreated in the presence or absence of DAPT (20 μM, 24 h) were stimulated with *H. pylori* (MOI 50, 24 h) and then co-cultured with splenic CD4^+^ T cells from syngeneic C57BL/6 mice for 72 h. The mRNA expression of characteristic transcription factors and cytokines of (**A**) Th1 (*Tbx21*, *Ifnγ*), (**B**) Th2 (*Gata3*, *Il4*), (**D**) Th17 (*Rorγt*, *Il17A*, *Il17F*) and (**E**) Treg (*Foxp3*, *Tgfβ*, *Il10*) was assessed using qRT-PCR. (**C**) The ratio of *Tbx21*/*Gata3* and (**F**) *Rorγt*/*Foxp3* is shown. Relative expression is normalized to β-Actin. The data are presented as the mean ± SD of three independent experiments. * *p* < 0.05, ** *p* < 0.01 and *** *p* < 0.001, **** *p* < 0.0001, N.S: no statistical difference.

**Figure 5:**
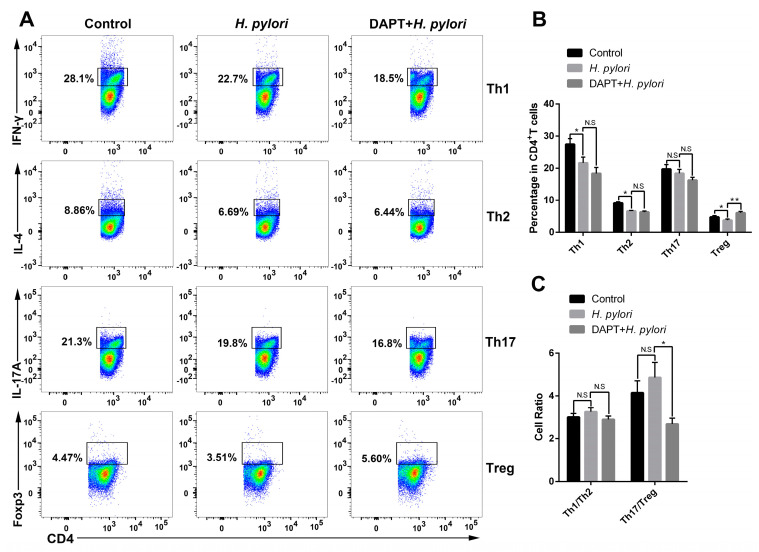
DAPT-pretreated BMDCs skewed Th17/Treg balance toward Treg. BMDCs pretreated in the presence or absence of DAPT (20 μM, 24 h) were stimulated with *H. pylori* (MOI 50, 24 h) and then co-cultured with splenic CD4^+^ T from syngeneic C57BL/6 mice for 72 h. Flow cytometric analyses were performed to measure the differentiation of CD4^+^ T cells. (**A**) Representative dot plots of Th1 (IFN-γ), Th2 (IL-4), Th17 (IL-17A) and Treg (Foxp3) are shown. (**B**) Percentages of Th1, Th2, Th17 and Treg and (**C**) the ratio of Th1/Th2 and Th17/Treg are shown. The data are presented as the mean ± SD of three independent experiments. * *p* < 0.05, ** *p* < 0.01, and N.S: no statistical difference.

Previous studies have shown that Notch signaling is involved in the regulation of DC activation, thereby affecting DC-induced CD4^+^ T cell differentiation in inflammatory and infectious diseases [16]. However, whether Notch signaling plays a role in DC-medicated Th differentiation during *H. pylori* infection has not been reported. To target Notch signaling, BMDCs were pretreated with or without DAPT before *H. pylori* infection, and then co-cultured with CD4^+^ T cells generated from syngeneic C57BL/6 mice. The expression of characteristic transcription factors and hallmark cytokines of Th1 (*Tbx21*, *Ifnγ*), Th2 (*Gata3*, *Il4*), Th17 (*Rorγt*, *Il17A* and *Il17F*) and Treg (*Foxp3*, *Tgfβ* and *Il10*) was determined using qRT-PCR. The results showed that *Rorγt*, *Il17A*, and *Il17F* were simultaneously up-regulated after *H. pylori* stimulation (Figure 4D), while the transcription factors and cytokines of other Th cells decreased (Figure 4A,B,E). Except for *Il17A*, DAPT pretreatment partially reversed the changes induced by *H. pylori*. In addition, the ratio of *Tbx21*/*Gata3* was down-regulated and the ratio of *Rorγ*t/*Foxp3* was up-regulated after *H. pylori* infection, whereas DAPT pretreatment reversed this change (Figure 4C,F). Subsequently, we performed flow cytometry to further detect the differentiation of CD4^+^ T cells (Figure 5A). In line with the mRNA level, the percentage of Th1, Th2, and Treg was down-regulated in *H. pylori*-pulsed BMDC group (Figure 5B). DAPT pretreatment elevated the percentage of Treg and had no effect on Th1 and Th2 (Figure 5B), which accounted for the decreased ratio of Th17/Treg (Figure 5C). Taken together, DAPT pretreatment in BMDCs shifted Th17/Treg balance toward Treg, revealing an important role of Notch signaling in CD4^+^ T cell differentiation during *H. pylori* infection.

## 4. Discussions

In recent decades, Notch signaling has been proven to be involved in the immune system, regulating T cell and B cell development, myeloid lineage commitment [38], and Th cell differentiation [39]. As for DCs, accumulating evidence has shown that Notch signaling affects DC development, maturation, and differentiation in vivo, [23,40] and is closely related to a variety of pathogen infections [16]. Meanwhile, Notch components are widely expressed in DCs, and different stimuli induce DCs to produce different expression profiles of receptors and ligands [24]. In this study, the mRNA level of all Notch receptors was elevated in BMDCs pulsed with *H. pylori*. As previously demonstrated, different ligands could activate distinct target programs through the same Notch receptor [41]. Among the five ligands of Notch signaling, each ligand has its own preference for Notch receptors, and the ligation of different ligands leads to different Notch intracellular domains (NICDs)’ translocation into the nucleus, resulting in activation of various downstream target genes [42]. Thus, different combinations of ligands and receptors have diverse effects. The Notch ligand Dll4 was increased in DCs infected with *mycobacteria* [43] and respiratory syncytial virus (RSV) [44]. Jagged1 was up-regulated after LPS [45] and Curdlan [46] stimulation. In accordance, our results showed that Dll4 and Jagged1 were elevated in *H. pylori*-infected BMDCs at mRNA level, but only Jagged1 showed a remarkable increase at protein level, consistent with our previous study in which the expression of Dll1, Dll4 and Jagged1 was significantly up-regulated in *H. pylori*-infected macrophages at the RNA level, but only Jagged1 was increased remarkably at the protein level [26], indicating that the mRNA and protein expression of Notch ligands are not always consistent. Therefore, we suppose that Jagged1 plays a crucial role in *H. pylori* infection.

Accumulating evidence has indicated that Notch signaling is involved in the modulation of cytokine secretion, thereby orchestrating the immune response [47]. Notch signaling was activated in *M. bovis* BCG pulsed-DCs, and enhanced the expression of pro-inflammatory cytokines TNF-α, IL-1β, and IL-6, whereas Notch signaling inhibitor DAPT hampered the production of TNF-α in plasmacytoid DCs and decreased the level of IL-10 in conventional DCs [48]. Similarly, DAPT reversed the elevation of TNF-α and IL-6 induced by *P. brasiliensis* [49]. In our study, the expression of IL-1β and IL-10 was increased in *H. pylori*-pulsed BMDCs, whereas DAPT treatment dramatically inhibited the production of IL-10, but further increased the expression of IL-1β, with little impact on TNF-α and IL-6. Unfortunately, there was no detectable IL-12, consistent with a previous study in which *H. pylori*-secreted factors inhibited IL-12 secretion of DCs [50]. The failure of TGF-β detection may be due to the low expression of TGF-β and the insufficient detection sensitivity of the kit. Under normal physiological conditions, pro-inflammatory and anti-inflammatory responses are in a state of dynamic balance. However, *H. pylori* infection breaks the balance, inducing high levels of pro-inflammatory cytokines. Meanwhile, excessive inflammatory responses lead to tissue damage and contribute to the pathogenesis of inflammatory disorders. In order to maintain immune homeostasis, the negative feedback regulation is also activated upon the initiation of pro-inflammatory response, which may explain the up-regulation of both pro-inflammatory cytokine IL-1β and anti-inflammatory cytokine IL-10 in our results. In addition, Gentle et al. found that Jagged1-mediated Notch signaling enhanced the expression of IL-10 and IL-2 in response to LPS. However, Jagged1 inhibited LPS-induced IL-12 secretion via a post-transcriptional mechanism. They further demonstrated that modulation of LPS-induced IL-10 and IL-2 was independent of γ-secretase-mediated canonical Notch signaling, indicating that the mechanisms vary in the regulation of different cytokines [35]. Thus, we supposed that the regulation of cytokines in *H. pylori*-infected DCs was dependent on different signals, and the cross-talk of multiple signaling pathways may be involved in the regulation of some cytokines [35,51]. Furthermore, DAPT pretreatment changed the profile of Notch receptors and ligands in BMDCs, which may partially influence the cytokine profile [36]. Taken together, these results suggest that Notch signaling is involved in the regulation of cytokines in *H. pylori*-infected BMDCs.

It has been proved that Notch signaling plays an important role in regulating the phenotype and maturation of DCs in many inflammatory and infectious diseases. For example, induction of Dll4 on DCs via concurrent stimulation of LPS and R848 (Toll like receptor 7/8 agonist) promoted the maturation of DCs characterized with high levels of CD80, CD86, CD40, CD103 and CD11b [52]. As for Jagged1, there are controversial views. One study showed that Jagged1 promoted the expression of maturation markers on DCs, including MHC-I, MHC-II, CD80, CD83, and CD86 [53], while another study suggested that Jagged1 decreased the levels of MHC-II, CD86 and CD40, inducing tolerogenic DCs [32]. In addition, there was a viewpoint that Jagged1 had no impact on DC maturation, but enhanced the expression of PD-L1 and OX40L (CD252) [54]. In the current study, *H. pylori* increased the expression of CD80, CD83, CD86, and MHC-II on BMDCs, in line with previous studies [55], while DAPT pretreatment partially reversed the increase in those surface markers. Therefore, we speculate that DAPT pretreatment may invalidate the function of Notch in DCs, resulting in the failure of transforming into fully mature DCs upon antigen stimulation.

DCs are key regulators of directing CD4^+^ T cell response in *H. pylori* infection [5], and the interaction between DCs and T cells involves multiple complex signals. Accumulating evidence has shown that Notch ligands on DCs, via binding to Notch receptors on T cells, influence T cell differentiation. For example, Dll4 promoted Th17 differentiation during *Mycobacteria* infection [56], and DAPT inhibited Th17 cell response [57]. In contrast, respiratory syncytial virus infection up-regulated the expression of Dll4 on DCs, resulting in an attenuated Th17 response [20]. In some studies, Jagged1 has been shown to be immunosuppressive. DCs expressing Jagged1 promote immunologic tolerance by inducing Treg differentiation [58]. However, another study indicated that DCs mediate Th17 polarization via Jagged1 activation [46]. Consistent with the latter report, our study showed that Jagged1 was up-regulated in *H. pylori*-pulsed BMDCs, leading to Th17 cell differentiation and Th1, Th2 and Treg cell impairment. Additionally, the ratio of *Tbx21*/*Gata3* decreased, while the ratio of *Rorγt*/*Foxp3* increased significantly in the co-culture of *H. pylori*-pulsed BMDCs and CD4^+^ T cells. However, DAPT pretreatment partially reversed those changes, except for *Il17A*. Furthermore, we analyzed the differentiation of CD4^+^ T cells via flow cytometry, and found that DAPT pretreated-BMDCs induced the differentiation of Treg, shifting the Th17/Treg balance to Treg.

In summary, our study demonstrated for the first time that Notch signaling plays an important role in the regulation of DC function and phenotype during *H. pylori* infection. Jagged1 may be a crucial regulator in this process, but its role in DCs and whether it is protective or pathogenic remains to be confirmed. As previously described, inhibition of Notch signaling may provide novel strategies for the prevention and treatment of infectious and inflammatory diseases, such as *tuberculosis* [59], chronic hepatitis [60] and Behcet’s disease [61]. However, extensive blockade of Notch signaling has obvious side effects, since Notch components are expressed in a variety of cells and tissues. We should pay more attention to this problem when exploring Notch signaling as a target for controlling *H. pylori* infection in the future.

## Figures and Tables

**Table 1:** Primer sequences for qRT-PCR in this study.

Species	Target Gene	Primer Sequence (5′→3′)	
Murine	*βActin*	Forward	GCAGGAGTACGATGAGTCCG
		Reverse	ACGCAGCTCAGTAACAGTCC
Murine	*Notch1*	Forward	ACGTAGTCCCACCTGCCTAT
		Reverse	CAGGTGCCCTGATTGTAGCA
Murine	*Notch2*	Forward	GTTGATCCCCGTCAGTGTGT
		Reverse	CAGGAGGCTGAAGTCGGTTT
Murine	*Notch3*	Forward	ACTCCTCCTCAGGGAGATGC
		Reverse	GTGGGGTGAAGCCATCAGG
Murine	*Notch4*	Forward	CCAGAGAGCTTCTGTGTGGA
		Reverse	CAGAAATCCAGGGGCACACT
Murine	*Dll1*	Forward	ACCAAGTGCCAGTCACAGAG
		Reverse	TCCATCTTACACCTCAGTCGC
Murine	*Dll3*	Forward	CTCCCGGATGCACTCAACAA
		Reverse	TGGAAGGGGCTGGTATGACA
Murine	*Dll4*	Forward	CTTTGGCAATGTCTCCACGC
		Reverse	ACTGCCGCTATTCTTGTCCC
Murine	*Jagged1*	Forward	GGGTCAGTTTGAGCTGGAGA
		Reverse	GTACGTATCACACTCGTCGC
Murine	*Jagged2*	Forward	GCCTCGTCGTCATTCCCTTT
		Reverse	AGCTCCTCATCTGGAGTGGT
Murine	*Il6*	Forward	TAGTCCTTCCTACCCCAATTTCC
		Reverse	TTGGTCCTTAGCCACTCCTTC
Murine	*Il12*	Forward	CAATCACGCTACCTCCTCTTTT
		Reverse	CAGCAGTGCAGGAATAATGTTTC
Murine	*Il1β*	Forward	TTCAGGCAGGCAGTATCACTC
		Reverse	GAAGGTCCACGGGAAAGACAC
Murine	*Tnfα*	Forward	GGTCACTGTCCCAGCATCTT
		Reverse	CTGTGAAGGGAATGGGTGTT
Murine	*Il10*	Forward	ATTTCCGATAAGGCTTGGCAA
		Reverse	GCTGGACAACATACTGCTAACC
Murine	*Tgfβ*	Forward	AGTGTGGAGCAACATGTGGAACT
		Reverse	AGCAGCCGGTTACCAAGGTA
Murine	*Tbx21*	Forward	AACACACACGTCTTTACTTTCCA
		Reverse	CGTATCAACAGATGCGTACATGG
Murine	*Ifnγ*	Forward	ACTGGCAAAAGGATGGTGAC
		Reverse	ACCTGTGGGTTGTTGACCTC
Murine	*Gata3*	Forward	CGAGATGGTACCGGGCACTA
		Reverse	GACAGTTCGCGCAGGATGT
Murine	*Il4*	Forward	TCTCGAATGTACCAGGAGCCATAT
		Reverse	AAGCACCTTGGAAGCCCTACAGA
Murine	*Rorγt*	Forward	CCGCTGAGAGGGCTTCAC
		Reverse	TGCAGGAGTAGGCCACATTACA
Murine	*Il17A*	Forward	TTTAACTCCCTTGGCGCAAAA
		Reverse	CTTTCCCTCCGCATTGACAC
Murine	*Il17F*	Forward	TGCTACTGTTGATGTTGGGAC
		Reverse	AATGCCCTGGTTTTGGTTGAA
Murine	*Foxp3*	Forward	CCCATCCCCAGGAGTCTTG
		Reverse	ACCATGACTAGGGGCACTGTA

**Table 2:** Antibodies used for Western blot in this study.

Antibodies	Dilution or Concentration	Source (Location)
Rabbit monoclonal anti-Jagged1	1:1000	Cell signaling, Danvers, MA, USA Cat. No. 2620S
Rabbit polyclonal anti-Dll4	1:1000	Affinity, San Francisco, CA, USA Cat. No. DF13221
Anti-Dll1 antibody	1:1000	Abcam, Cambridge, UK No. ab84620
β-Tubulin (9F3) Rabbit mAb	1:1000	Cell signaling, Danvers, MA, USA Cat. No. 2128S
Peroxidase conjugated Goat anti-Rabbit IgG	1:10,000	Fude BioTech, Hangzhou, China Cat. No. FDR007

## Data Availability

Data are available on request.
